# Infarct myocardium tissue heterogeneity assessment using pre-contrast and post-contrast T1 maps acquired with Modified Look-Locker Inversion Recovery (MOLLI) imaging

**DOI:** 10.1186/1532-429X-14-S1-P263

**Published:** 2012-02-01

**Authors:** Zhong Chen, Tobias Voigt, Andrea Wiethoff, Siobhan Crichton, David Murday, Anoop Shetty, Aldo Rinaldi, Eike Nagel, Valentina O Puntmann, Tobias Schaeffter, Reza Razavi

**Affiliations:** 1 Imaging Sciences & Biomedical Engineering, Kings College London, London, UK; 2 Southampton University Hospitals NHS Trust, Southhampton, UK

## Summary

T1 relaxation-time mapping allows direct myocardial signal quantification and therefore enables true quantitative characterisation of myocardial tissue heterogeneity. Differences between healthy myocardium and scarred tissues can be reliably distinguished from the R1 values derived from pre-contrast T1 maps. In patients with scarred tissues, ΔR1 value derived from both the pre- and the post-contrast T1 maps provides better distinction between grey zone and scar core than either pre-contrast or post-contrast R1 value alone.

## Background

Cardiac magnetic resonance imaging with late gadolinium enhancement (LGE-CMR) has been the standard tool for assessing regional fibrosis. Tissue heterogeneity quantification by traditional signal-intensity (SI-) based methods is not without limitations. T1 relaxation-time mapping allows direct myocardial signal quantification and therefore enables true quantitative characterisation of myocardial tissue heterogeneity. We aim to explore tissue heterogeneity assessment using T1 maps generated with the modified Look Locker (MOLLI) sequence in patients with previous myocardial infarct.

## Methods

Seven patients with ischaemic infarct history underwent left ventricular scar assessment with standard inversion-recovery gradient-echo sequence 10 minutes post-contrast (gadolinium-DTPA, 0.2mmol/kg) in short axis (voxel size 1.8x1.8x8mm) on a 1.5T scanner (Philips Healthcare, Best, Netherlands). Three separate slices of MOLLI images were taken pre- and 20 minutes post-contrast in the same geometry. SI-based tissue heterogeneity assessment on the LGE-CMR images identified the regions of remote healthy myocardium (n=21), scar core (n=21) and grey zone (n=21); 2-standard-deviation SI from the Remote<Grey<Core defined by the full-width-half-maximum method. The corresponding regions-of-interest were identified on the pre- and post-contrast T1 maps. T1 and R1 values (1x103/T1) for the respective regions were analysed. Statistical analyses were performed using Mann-Whitney U and Wilcoxon tests.

## Results

**Figure 1:**
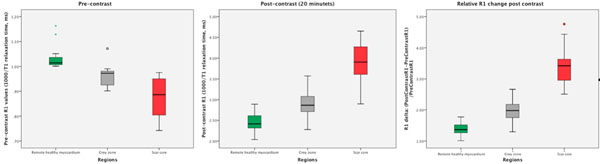
Effect of contrast on R1 values (1x103/T1 relaxation time) and the relative changes, ΔR1, for regions of remote healthy myocardium, grey zone and scar core; n=21, respectively

The T1 relaxation-times for each region were significantly different on both the pre- and the post-contrast T1 maps; p<0.05. At 20 minutes post-contrast, the T1 values were significantly reduced in all regions with the greatest reduction seen in scar core; p<0.05. The R1 and the ΔR1 (defined as (PostContrastR1-PreContrastR1)/PreContrastR1) for each region are plotted in Figure 1.

**Figure 2:**
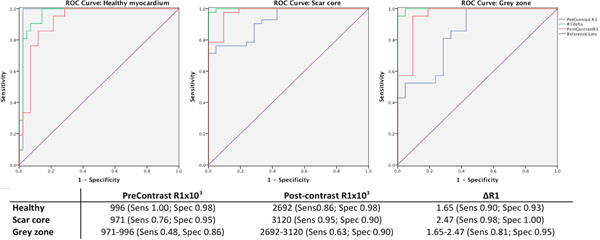
ROC curve analysis of remote healthy myocardium, scar core and grey zone assessment by pre-contrast R1, post-contrast R1 and ΔR1; n = 21, respectively.

ROC curve analysis showed that the native pre-contrast R1 provided the best prediction for healthy myocardium whereas the ΔR1 provided better prediction for scar core and grey zone than either the pre-contrast or the post-contrast R1 value alone (Figure 2). A cut-off of pre-contrast R1 of 0.996 provided the best distinction for the healthy myocardium; a ΔR1 of 2.47 provided the best distinction between the scar core and the grey zone.

## Conclusions

T1 maps acquired from MOLLI sequence allow quantitative assessment of tissue heterogeneity. Differences between healthy myocardium and scarred tissues can be reliably distinguished from the R1 values derived from pre-contrast T1 maps. Potentially, patients without scarred myocardium do not need post-contrast imaging. In patients with scarred tissues, ΔR1 value derived from both the pre- and the post-contrast T1 maps provides better distinction between grey zone and scar core than either pre-contrast or post-contrast R1 value alone.

## Funding

N/A

